# Supplementary material for: Metabolomic Profile of Different Dietary Patterns and Their Association with Frailty Index in Community-Dwelling Older Men and Women
Source: Nutrients. 2022 May 27;14(11):2237. doi: 10.3390/nu14112237 (PMC9182888; doi:10.3390/nu14112237)
Supplement: Supplementary file 1 [file nutrients-14-02237-s001.zip › BLSA_metabolomics_diet_FI_Supplemental_tables_figures.pdf]

Table S1. Components of dietary patterns.

MIND Diet Score		Mediterranean Diet Score		AHEI Diet Score	
MIND Diet Components		Mediterranean Diet Components		AHEI Diet Components	
Frequency or Specified	Max score	Serving or Gram (sex-specific median)	Max score	Serving or Specified	Max score
Green Leafy Vegetables≥6/wk	1	Vegetables ≥Median	1	Vegetables≥5	10
Other Vegetables≥1/day	1				
Berries≥2/wk	1	Fruits and Nuts ≥Median	1	Fruits≥4	10
Nuts≥5/wk	1			Nuts and Legumes≥1	10
Beans>3/wk	1	Legumes ≥Median	1		
Olive Oil=Primary oil used	1				
Butter, Margarine<1 T/d	1				
Cheese< 1/wk	1	Dairy <Median	1		
Whole Grains≥3/d	1	Whole Grains ≥Median	1	Whole Grains, ≥90g for men, ≥75g for women	10
Fish (not fried)≥1/wk	1	Fish ≥Median	1		
Poultry (not fried)≥2/wk	1				
Red Meat and products< 4/wk	1	Red and Processed Meat <Median	1	Red and Processed Meat=0	10
Fast Fried Foods<1/wk	1				
Pastries & Sweets<5/wk	1			Sugar-sweetened Beverage and Fruit Juice=0	10
Wine, glass=1/d	1	Ethanol, 10-50g for men, 5-25g for women	1	Alcohol, 0.5-2 drinks for men, 0.5-1.5 drinks for women	10
		Ratio of Monounsaturated to Saturated Fats ≥Median	1	Polyunsaturated Fats≥10% of energy	10
				trans Fat≤0.5% of energy	10
				Long-chain Fats (EPA+DHA)≥250mg	10
				Sodium, lowest decile	10
Total MIND Diet Score	15	Total Med Diet Score	9	Total AHEI Diet Score	110

Table S2. Algorithm for construction of frailty index

Deficit components	Cut-offs
Anemia	0 = No, 1= Yes
Cancer	
Chronic kidney disease	
Chronic obstructive pulmonary disease	
Cognitive impairment	
Congestive heart failure	
Depression	
Diabetes	
Heart Disease	
Hip replacement	
Hypertension	
Lower body joint disease	
Parkinson`s disease	
Peripheral arterial disease	
Stroke	
Walking up/down staircase ten steps	0 = No difficulty/very easy/somewhat easy<br>0.33 = With a little or some difficulty<br>0.66 = With a lot of difficulty<br>1 = Unable to do"
Lifting/carry 10lbs	
Getting in/out of bed	0= No difficulty and no assistance<br>0.33= With difficulty but no assistance<br>0.66= With difficulty with assistance<br>1= Unable to do
Bathing and showering	
Dressing	
Eating	
Toileting	
Walking across small room	
Heavy housework	0 = No difficulty or don't do (unrelated to health)<br>0.33 = A little or some difficulty or don't do because of health<br>0.66 = "A lot" difficulty doing<br>1 = unable to do
Food preparation	
Shopping	
Using telephone	
Medication use	
Managing finances	
Continence	0 = No<br>0.5 = Either urinary or fecal incontinence<br>1 = Both urinary and fecal incontinence
Self-rated health	Excellent = 0, very good = 0.25, good = 0.50, fair = 0.75, poor = 1
Feel depressed (CES-D)	0 = Rarely<br>0.33 = Some of the time<br>0.66 = Occasionally or moderately<br>1 = Most or all the time
Feel everything is an effort (CES-D)	
Could not get going (CES-D)	
Feel lonely (CES-D)	
Feel happy (CES-D)	0 = Most or all the time<br>0.33 = Occasionally or moderately<br>0.66 = Some of the time<br>1 = Rarely
Orientation time (MMSE)	0 = no error<br>0.50 = 1 error<br>1 = 2 or more errors
Orientation place (MMSE)	
Attention (MMSE)	
Recall (MMSE)	0 = three correct, 0.50 = two correct, 1 = one or none correct
Weight loss 5% in past year	0 = No, 1= Yes
Low physical activity	0 = No, 1= Yes
Slow gait speed	0 = Normal, 1=lowest quintile, stratified sex adjusted for height
Low grip strength	0 = Normal, 1=lowest quintile, stratified by sex adjusted BMI

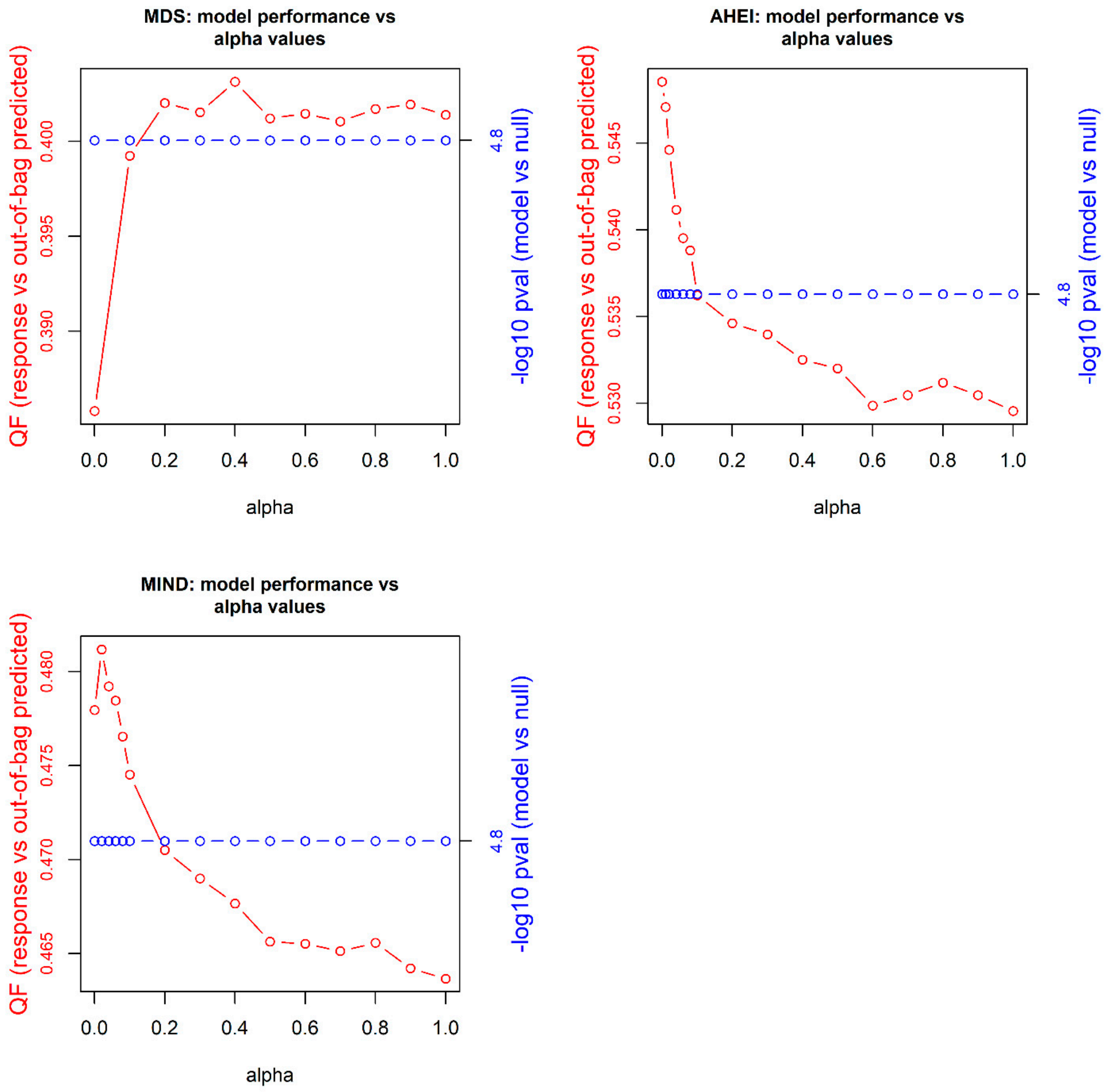

Figure S1. Cross-validated performance of prediction models across alpha values. The figure displays results from elastic net regression models runs. Quality function (QF) or -log10 of statistical significance (p-value of model vs null) across difference alpha values.

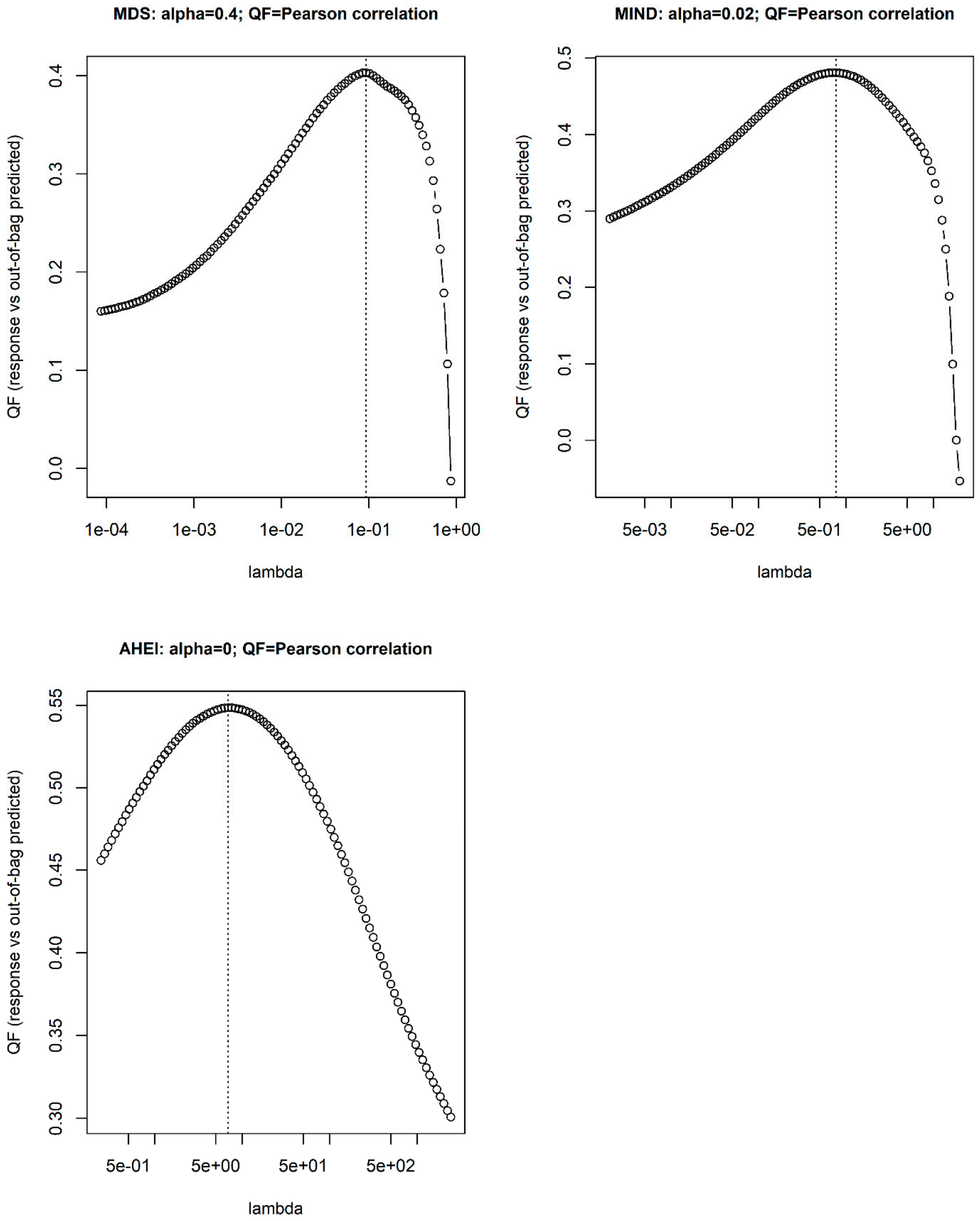

Figure S2. Selection of optimal penalty parameter λ by quality function (QF) maximization at the chosen mixing parameter alpha values for each diet score response.

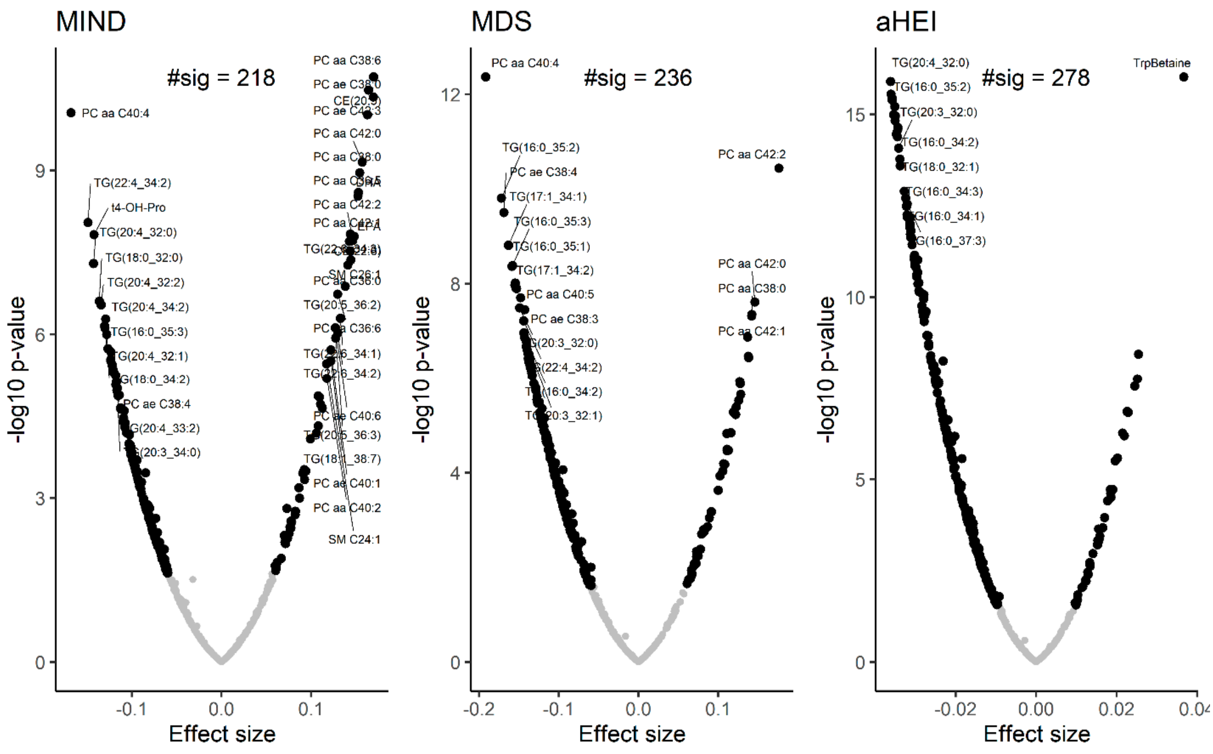

Figure S3. Associations between plasma metabolites and dietary patterns.

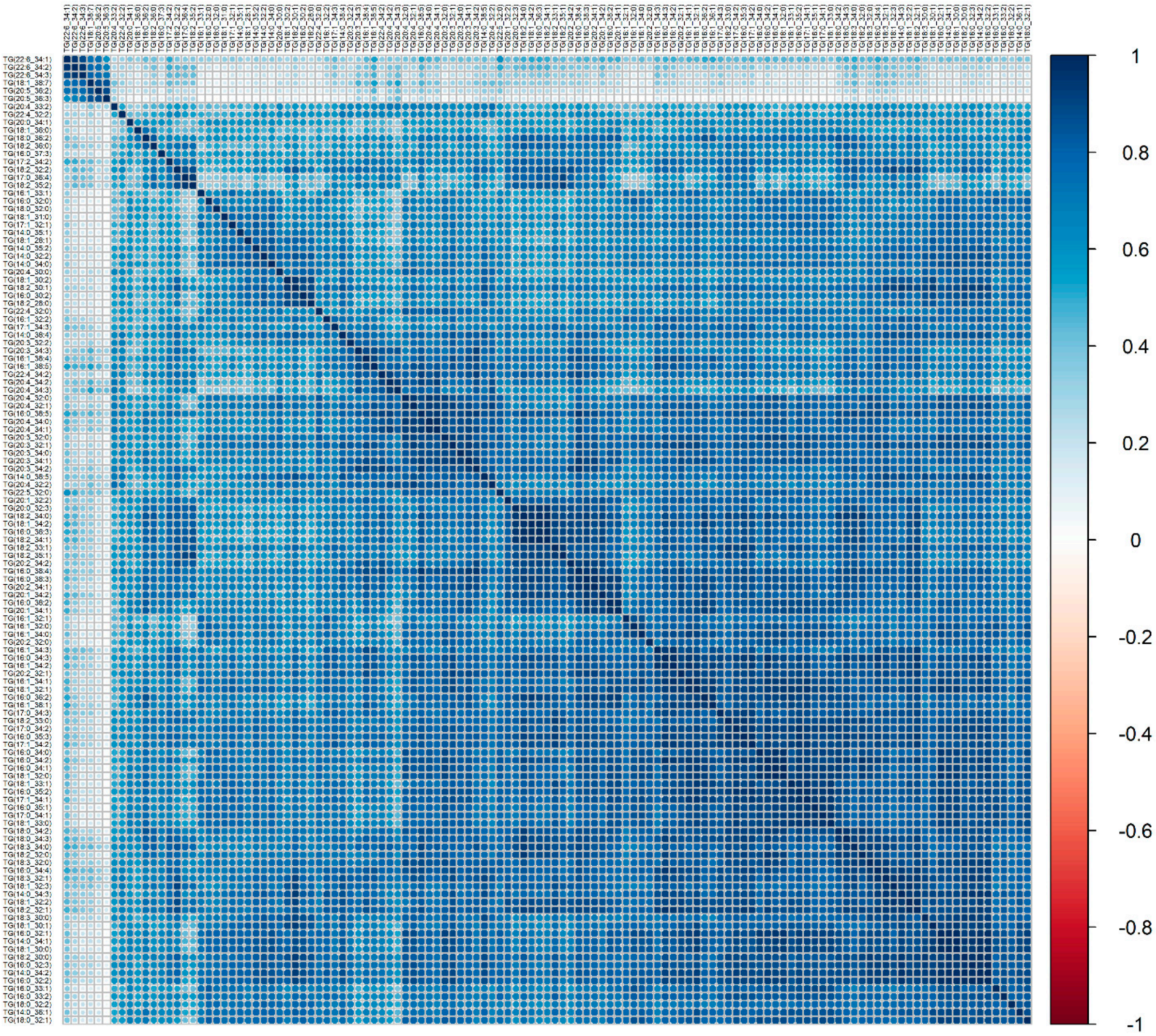

Figure S4. Correlation coefficients of diet-associated triglycerides.

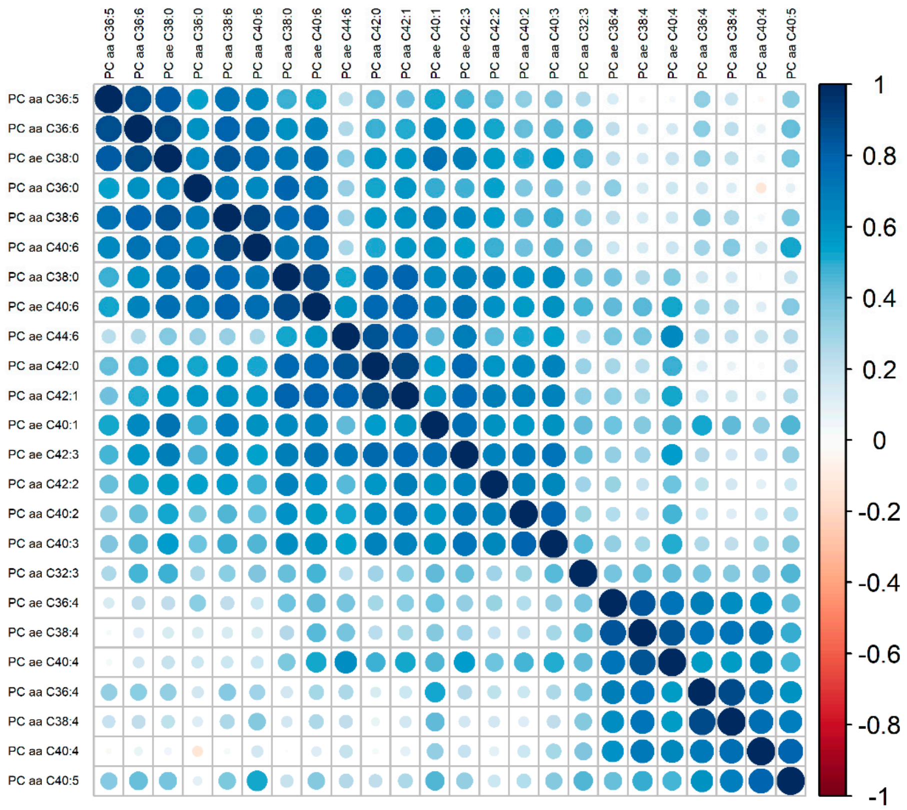

Figure S5. Correlation coefficients of diet-associated phosphatidyl-choline.
